# Effects of cross-bridge compliance on the force-velocity relationship and muscle power output

**DOI:** 10.1371/journal.pone.0190335

**Published:** 2017-12-28

**Authors:** Axel J. Fenwick, Alexander M. Wood, Bertrand C. W. Tanner

**Affiliations:** Department of Integrative Physiology and Neuroscience, Washington State University, Pullman, Washington, United States of America; University of Michigan, UNITED STATES

## Abstract

Muscles produce force and power by utilizing chemical energy through ATP hydrolysis. During concentric contractions (shortening), muscles generate less force compared to isometric contractions, but consume greater amounts of energy as shortening velocity increases. Conversely, more force is generated and less energy is consumed during eccentric muscle contractions (lengthening). This relationship between force, energy use, and the velocity of contraction has important implications for understanding muscle efficiency, but the molecular mechanisms underlying this behavior remain poorly understood. Here we used spatially-explicit, multi-filament models of Ca^2+^-regulated force production within a half-sarcomere to simulate how force production, energy utilization, and the number of bound cross-bridges are affected by dynamic changes in sarcomere length. These computational simulations show that cross-bridge binding increased during slow-velocity concentric and eccentric contractions, compared to isometric contractions. Over the full ranges of velocities that we simulated, cross-bridge cycling and energy utilization (i.e. ATPase rates) increased during shortening, and decreased during lengthening. These findings are consistent with the Fenn effect, but arise from a complicated relationship between velocity-dependent cross-bridge recruitment and cross-bridge cycling kinetics. We also investigated how force production, power output, and energy utilization varied with cross-bridge and myofilament compliance, which is impossible to address under typical experimental conditions. These important simulations show that increasing cross-bridge compliance resulted in greater cross-bridge binding and ATPase activity, but less force was generated per cross-bridge and throughout the sarcomere. These data indicate that the efficiency of force production decreases in a velocity-dependent manner, and that this behavior is sensitive to cross-bridge compliance. In contrast, significant effects of myofilament compliance on force production were only observed during isometric contractions, suggesting that changes in myofilament compliance may not influence power output during non-isometric contractions as greatly as changes in cross-bridge compliance. These findings advance our understanding of how cross-bridge and myofilament properties underlie velocity-dependent changes in contractile efficiency during muscle movement.

## Introduction

During concentric muscle contraction, the force generated during shortening decreases non-linearly as the shortening velocity increases. Reciprocally, shortening velocity of a concentrically-contracting muscle increases as the resistive load decreases, until the muscle reaches its maximal unloaded shortening velocity (*V*_*max*_). Conversely, forced-lengthening of a contracting muscle (i.e. eccentric contraction) at loads that are greater than that of isometric contraction results in a rapid rise in force [1]. This classic signature of muscle contraction is known as the force-velocity relationship [2,3]. However, muscles utilize more energy during concentric contractions than during either isometric or eccentric contractions, representing an observation from nearly a century ago that is called the Fenn effect [2,4]. While no external work is performed by the muscle at *V*_*max*_, the continued consumption of chemical energy means that the system efficiency approaches zero [5]. These observations suggest that contracting muscles must balance power output and efficiency during lengthening and shortening transients that facilitate locomotion. However, the molecular mechanisms that underlie these coupled relationships of force, velocity, and energy utilization remain unclear.

Mathematical models and computational simulations of muscle contraction are useful tools for predicting and estimating specific parameters that underlie muscle function throughout a contraction. Since A.F. Huxley proposed the sliding filament hypothesis [6], mass action kinetic models of actin-myosin cross-bridge interactions have provided important insights into muscle contraction. However, mass-action models of actin-myosin interactions that describe system behavior via ordinary differential equations intrinsically assume that cross-bridges operate as independent force generators and do not explicitly consider spatial nor mechanical properties of the myofilaments that may influence contraction [6–14]. Given that myosin cross-bridges never actually operate in isolation in a cell, the impacts of spatial and mechanical protein coupling on ensemble myosin cross-bridge activity are difficult to predict mathematically or clearly separate and define with well-defined experiments. Nonetheless, the development of spatially-explicit sarcomeric models have provided significant insights about the spatial and mechanical mechanisms of coupling that underlie muscle contraction [15–24]. This class of models also enables simulations of isometric and dynamic muscle contraction where filaments actually slide past each other, while tracking details such as the number of activated thin-filament sites or bound cross-bridges, myosin ATPase rates, individual and ensemble-average cross-bridge forces that contribute to sarcomeric tension development, etc. As nearly 70% of muscle compliance resides in the thick and thin filaments [25–29], these models have illustrated how changing the mechanical characteristics of the filaments and cross-bridges profoundly influences the levels of cross-bridge binding, force production, and ATP utilization throughout the compliant myofilament lattice. This significant compliance implies that cross-bridges do not operate independently while generating force, yet the potential effects of cross-bridge stiffness and filament compliance on ensemble cross-bridge activity during lengthening and shortening have received limited study [15,23].

**Fig 1:**
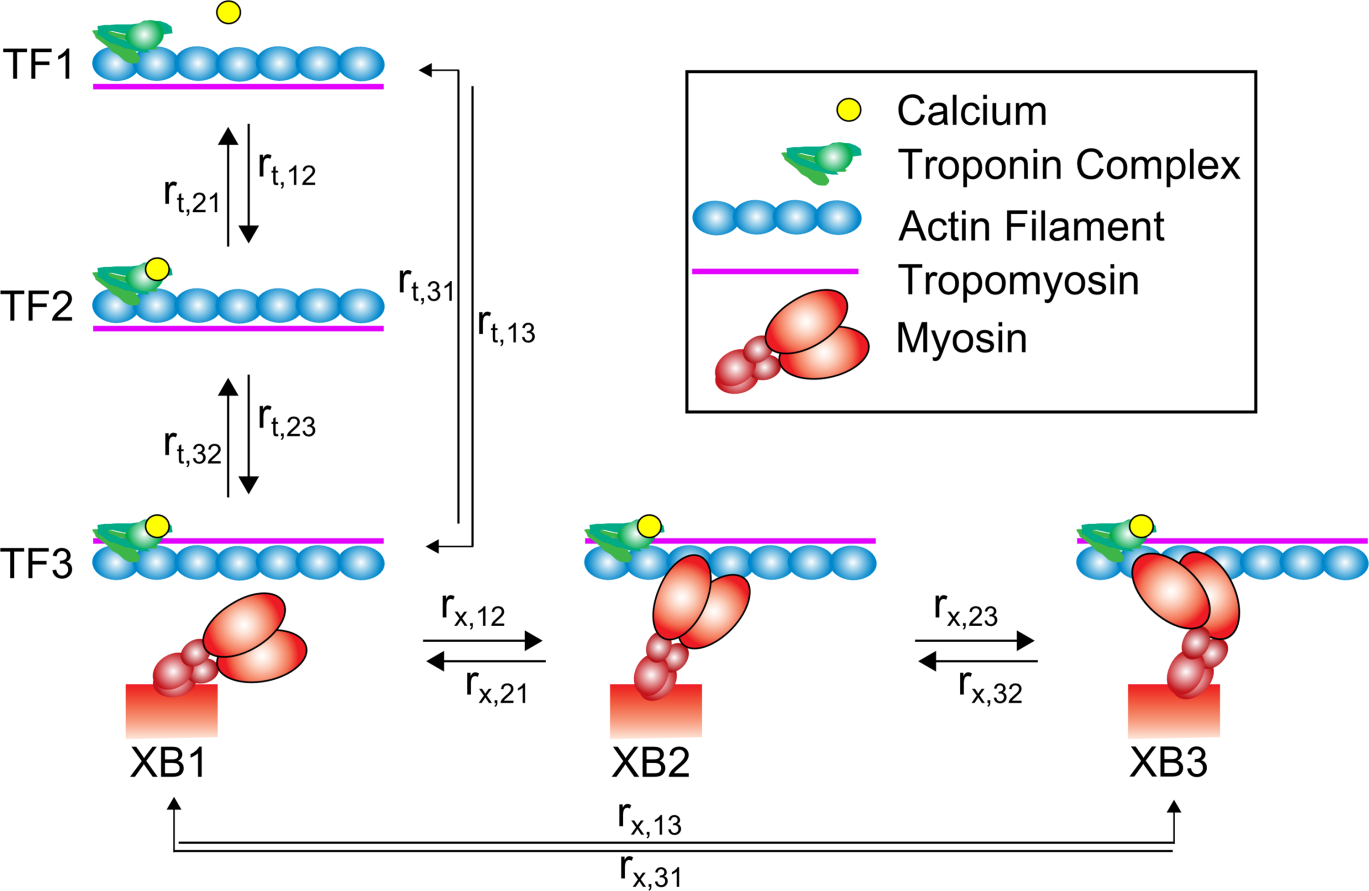
Overview of modeled kinetic transitions. Our computational model combines two three-state cycles to simulate activation of thin-filament regulatory units (r_t,ij_) and the cycling of cross-bridges (r_x,ij_). Thin-filament activation is described by an initial state where calcium is unbound from the troponin complex and tropomyosin blocks the actin-target zone (TF1), a second state where calcium is bound to troponin but tropomyosin remains in a blocking conformation (TF2), and a final state where the calcium activation of troponin shifts tropomyosin and reveals the actin-target zone (TF3). Following TF3, cross-bridge binding occurs in three states: an initial unbound state (XB1), a low-force bearing, pre-power stroke state (XB2), and a high-force bearing, post-power stroke state (XB3).

**Fig 2:**
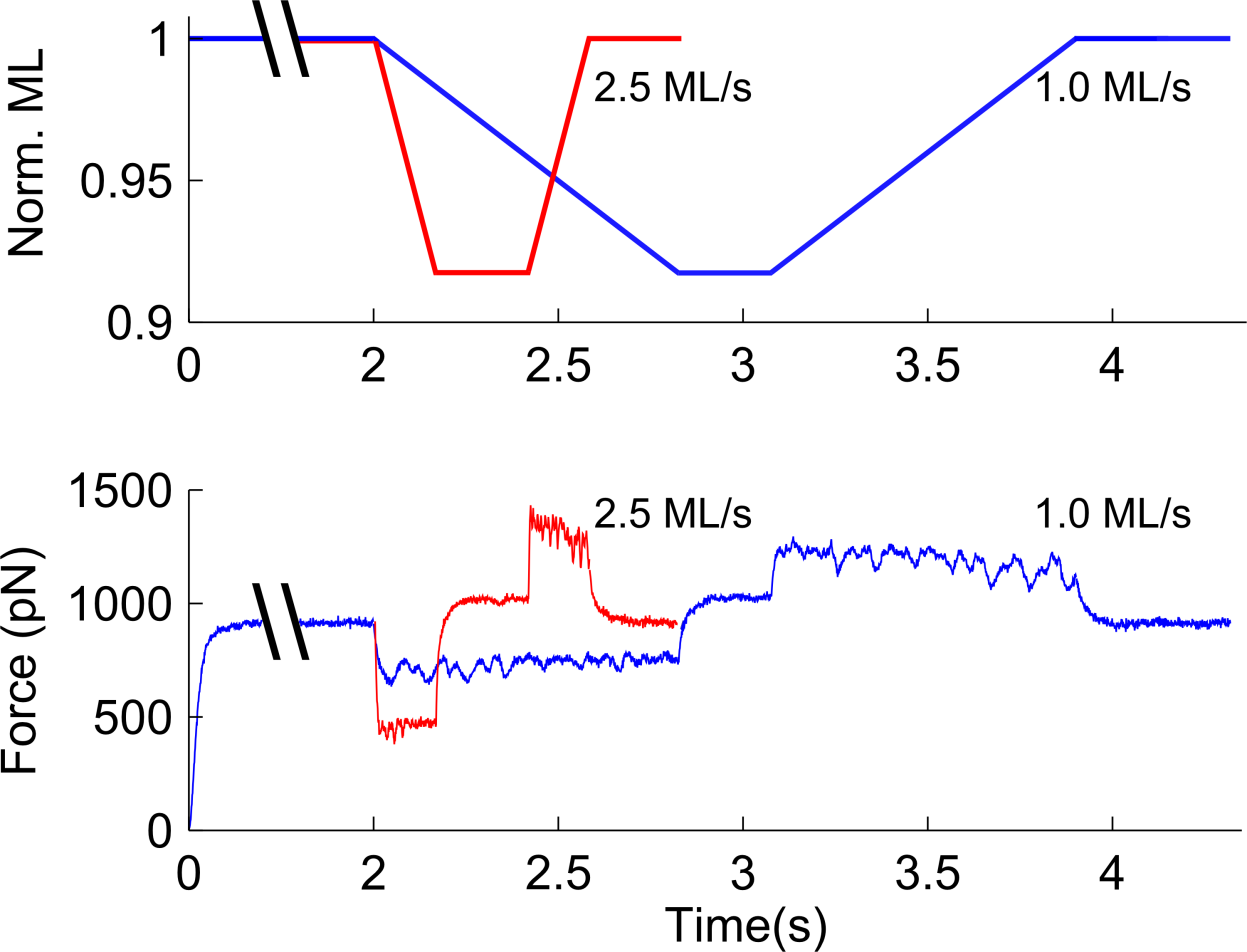
Protocol for simulating length transients. A) Following activation, the half-sarcomere was shortened by 100 nm (~10% half-sarcomere length) at a velocity between 0.1 and 4.5 muscle lengths per second (ML/s). Sarcomeres were held at this new length for 250 ms and then returned to the initial length at the same velocity they were shortened. B) Force traces over this period show decreased force during shortening and increased force during lengthening, with a dependence on the velocity of shortening and lengthening. Example traces from 0.5 and 1.5 ML/s are shown here, superimposed.

Here we utilize spatially-explicit, multi-filament models of Ca^2+^-regulated force production in a half-sarcomere to simulate muscle contraction during shortening and lengthening. Thin-filament activation and cross-bridge cycling kinetics are represented as two coupled three-state models (Fig 1). These models build upon an earlier model described by Tanner et al. [20], with the addition of a non-linear elastic element to represent titin and provide a length-dependent passive tension response [30]. This addition enabled us to simulate how force production and myosin kinetics were influenced by shortening and lengthening transients applied to the half-sarcomere (Fig 2). Together, these model developments facilitated simulations that relate changes in force production, shortening velocity, and power output with respective estimates of cross-bridge number, force borne per cross-bridge, and ATPase activity which are difficult or impossible to parse from empirical observations alone.

An additional benefit of these numerical experiments is the ability to directly manipulate mechanical characteristics of the sarcomere, such as cross-bridge or myofilament compliance. For example, recent studies have shown that the stiffness (inverse of compliance) of myosin may be regulated through phosphorylation of the regulatory light chain, which results in a prolonged myosin duty cycle and slower contractile velocity [31,32]. These findings suggest that the behavior and efficiency of contraction dictated by the force-velocity relationship may be especially sensitive to altered cross-bridge compliance. Huxley and Tideswell estimated cross-bridge stiffness to be around 2 pN nm^-1^ [33], though various single-molecule measurements have suggested stiffness values as low as 0.5 pN nm^-1^ and as high as 3.3 pN nm^-1^ [34–37]. Here, we show how the kinetics and efficiency of isometric and non-isometric contractions respond to variations in cross-bridge stiffness, illustrating potential benefits and costs to muscle work and power output at the molecular and sarcomeric levels as cross-bridge stiffness changes.

## Results

**Fig 3:**
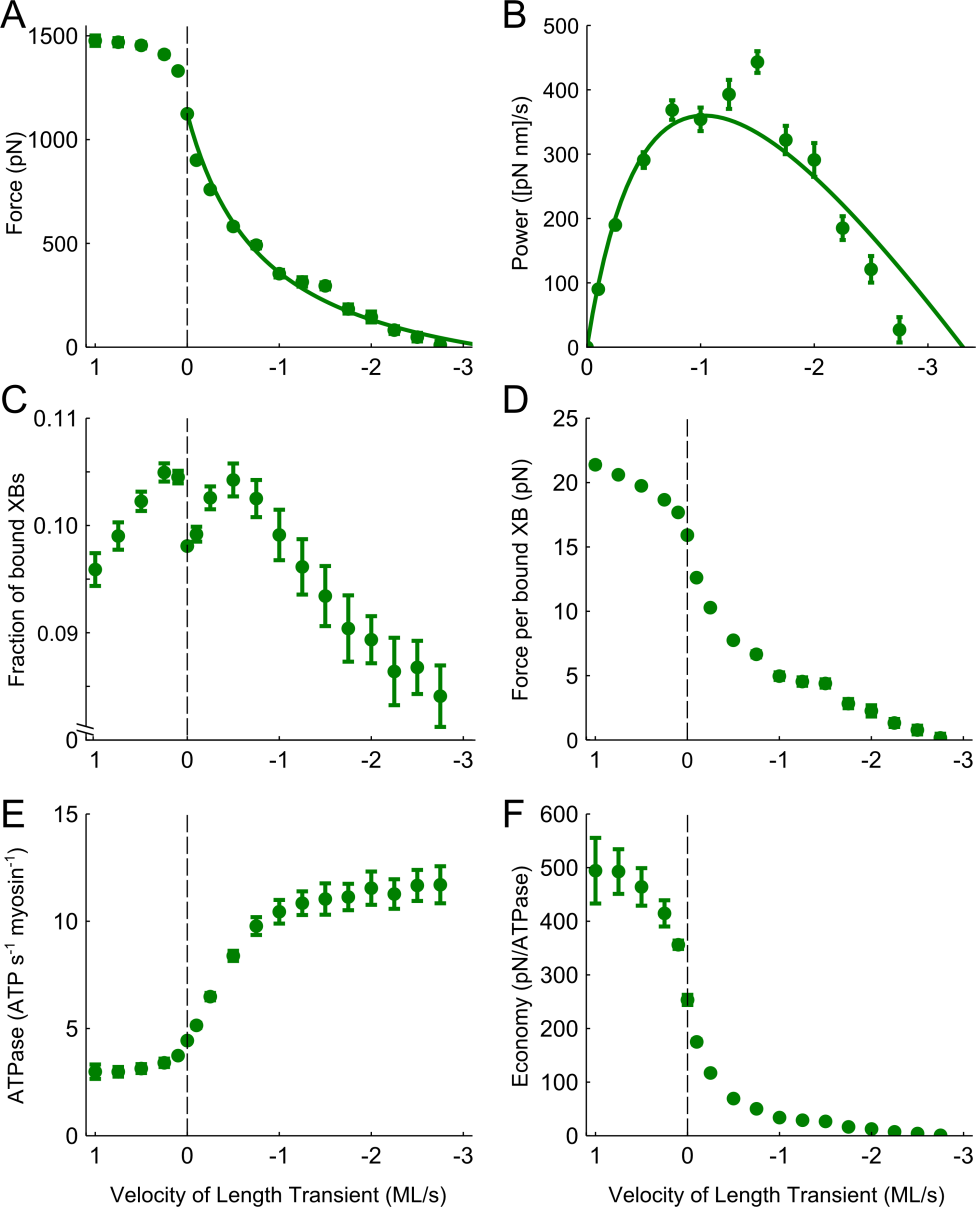
Velocity-dependent measurements with XB stiffness fixed at 3 pN nm^-1^. A) Active force increased during lengthening and decreased during shortening, reaching maximal unloaded shortening around 3 ML/s. B) Power was calculated during the shortening transient as the product of force and velocity, producing a hyperbolic curve with peak power around 1 ML/s. C) The fraction of cross-bridges bound to the thin-filament was greatest during relatively slow length transients in either direction. D) The ratio of total force distributed amongst the bound cross-bridges increased during lengthening and decreased during shortening. E) The amount of ATP consumed for the total number of myosin heads in the system decreased slightly during the lengthening transients and increased with shortening velocity. F) The ratio of force to ATPase activity was greatest during lengthening and lowest during unloaded shortening.

Force production, cross-bridge binding, and ATP utilization were influenced by both lengthening and shortening of the sarcomere in a velocity-dependent manner. In accordance with the typical force-velocity relationship [2], lengthening increased force production from the isometric value and shortening decreased force production from the isometric value (Fig 3A). During lengthening, force increased with increasingly faster lengthening velocities up to ~0.75 muscle lengths/second (ML/s), above which force plateaued at values 40–50% greater than isometric force. As shortening velocity increased, force decreased in a hyperbolic manner until the maximal unloaded shortening velocity reached -3.31 ML/s (Fig 3A, Table 1). Power output, calculated as the product of force and shortening velocity, was characterized as a parabolic curve with a maximal peak (*V*_*opt*_) around -1 ML/s (Fig 3B, Table 1), as shown by the curve-fit that was translated from the force-velocity relationship (Eq 2; solid lines in Fig 3A and 3B).

**Table 1:** Summary of force and power data with varying cross-bridge stiffness.

XB stiffness (pN/nm)	Isometric force (pN)	*V*_*max*_ (ML/s)	Peak Power (pN nm s^-1^)	*V*_*opt*_ (ML/s)
1	869.0±2.8	1.65	513.9	1.60
3	1123.6±3.5	3.31	359.9	1.05
10	951.7±4.5	4.22	280.2	0.70

During these shortening and lengthening transients there were a number of velocity-dependent influences on myosin cross-bridge behavior that underlie these force-velocity predictions (Fig 3C–3F). The fraction of myosin cross-bridges that were formed between thick and thin-filaments increased during slow lengthening and slow shortening velocities (±0.75 ML/s), compared to the fraction of cross-bridges bound during isometric contractions. At shortening and lengthening transients faster than ±1 ML/s, however, cross-bridge binding decreased below the isometric value (Fig 3C). The average force borne by individual cross-bridges increased during lengthening and decreased during shortening (Fig 3D), with a comparable profile to that of the force-velocity relationship in Fig 3A. ATPase activity, represented as the amount of ATP consumed for the total number of myosin heads in the system, decreased slightly during the lengthening transients (Fig 3E). Conversely, ATPase activity significantly increased with shortening velocity. Dividing force production by ATPase activity provides an estimate of the economy of force and shows more economical force production during lengthening and less economical force production during shortening (Fig 3F).

**Fig 4:**
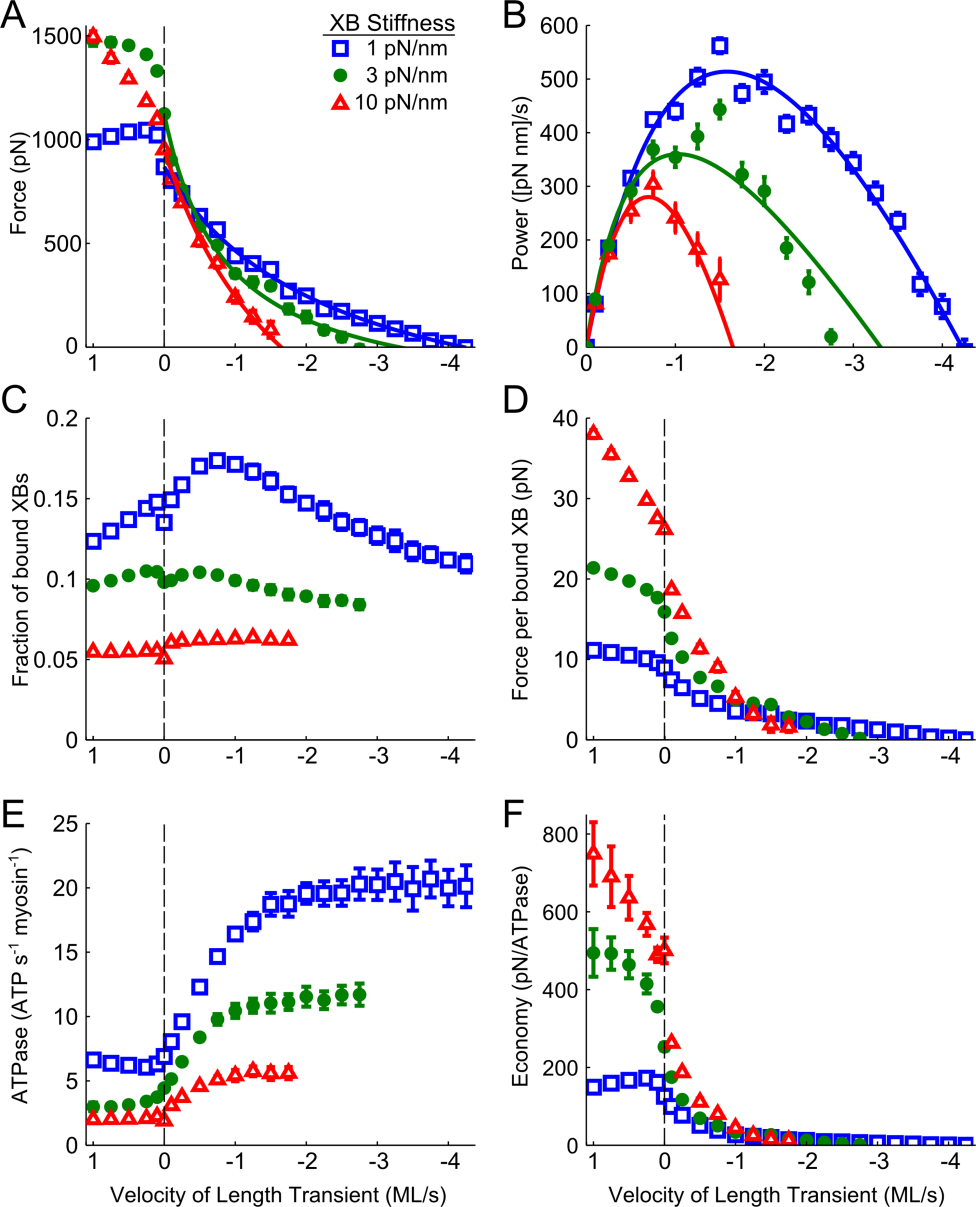
Velocity-dependent measurements as a function of XB stiffness. A) Decreased XB stiffness resulted in the maintenance of more force during sarcomere shortening and a faster unloaded shortening velocity, while the converse occurred with increased stiffness. B) Decreased XB stiffness resulted in greater power generation, with the peak power shifted towards a faster shortening velocity. C) Decreased XB stiffness resulted in a greater fraction of bound XBs, particularly during slow shortening transients. D) Decreased XB stiffness resulted in less force per bound XB, though force was maintained at faster velocities in sarcomeres with more stiff XBs. E) Decreasing XB stiffness significantly increased the total ATPase activity during isometric and non-isometric contractions. F) Decreasing XB stiffness lowered the economy of force production during both lengthening and shortening transients compared to sarcomeres with greater XB stiffness (up to their respective unloaded shortening velocities).

Force production, cross-bridge binding, and ATP utilization were also influenced by the stiffness of myosin cross-bridges (XBs). Increasing XB stiffness to 10 pN/nm or decreasing XB stiffness to 1 pN/nm (vs. the 3 pN/nm XB stiffness shown in Fig 3) both lowered isometric force (by 15% and 23%, respectively) (Fig 4A). As lengthening velocity increased, force production approached a plateau with a rate and magnitude that varied with XB stiffness; specifically, force plateaued around 2500 pN above 4 ML/s (data not shown) when XB stiffness was 10 pN/nm and force plateaued around 1000 pN above 0.25 ML/s when XB stiffness was 1 pN/nm. Compared to the 3 pN/nm data, increased XB stiffness resulted in less force production at shortening velocities faster than -1.25 ML/s and slowed maximal unloaded shortening velocity to -1.65 ML/s (Table 1). Decreased XB stiffness resulted in more force production at shortening velocities faster than 1.5 ML/s and raised the maximal unloaded shortening velocity to -4.22 ML/s. As a result of these differences in the force-velocity relationship, increasing cross-bridge stiffness led to lower power output with a slower *V*_*opt*_ (Fig 4B). Conversely, reducing cross-bridge stiffness led to greater power generation, which peaked at a faster shortening velocity.

As cross-bridge stiffness increased from 3 to 10 pN/nm the fraction of bound XBs decreased across the entire range of velocity transients (Fig 4C), and the fraction of bound XBs increased as XB stiffness decreased to 1 pN/nm. For all values of cross-bridge stiffness, relatively slow shortening or lengthening velocity increased the fraction of bound cross-bridges from their isometric value, yet the relative increase in cross-bridge fraction varied with cross-bridge stiffness and velocity (Fig 4C). These differences in XB binding resulted in greater force borne per bound XB when cross-bridge stiffness increased, and less force per bound XB when cross-bridge stiffness decreased. These effects of XB stiffness were greatest during lengthening (Fig 4D). Compared to 3 pN/nm XB stiffness values, isometric ATPase activity decreased by 57% as XB stiffness increased to 10 pN/nm, and increased by 55% as XB stiffness decreased to 1 pN/nm. Regardless of stiffness, ATPase remained relatively insensitive during lengthening transients. However, ATPase activity increased with increases in the shortening velocity, with the largest ATPase values occurring during maximal shorting when XB-stiffness was decreased to 1 pN/nm (Fig 4E). Isometric force production was more economical with the stiffer XBs (98% increase), and was less economical with decreased XB-stiffness (50% decrease; Fig 4F). These XB stiffness-dependent differences in the economy of force were amplified during lengthening, and dampened during shortening, eventually disappearing at the fastest shorting velocity.

**Fig 5:**
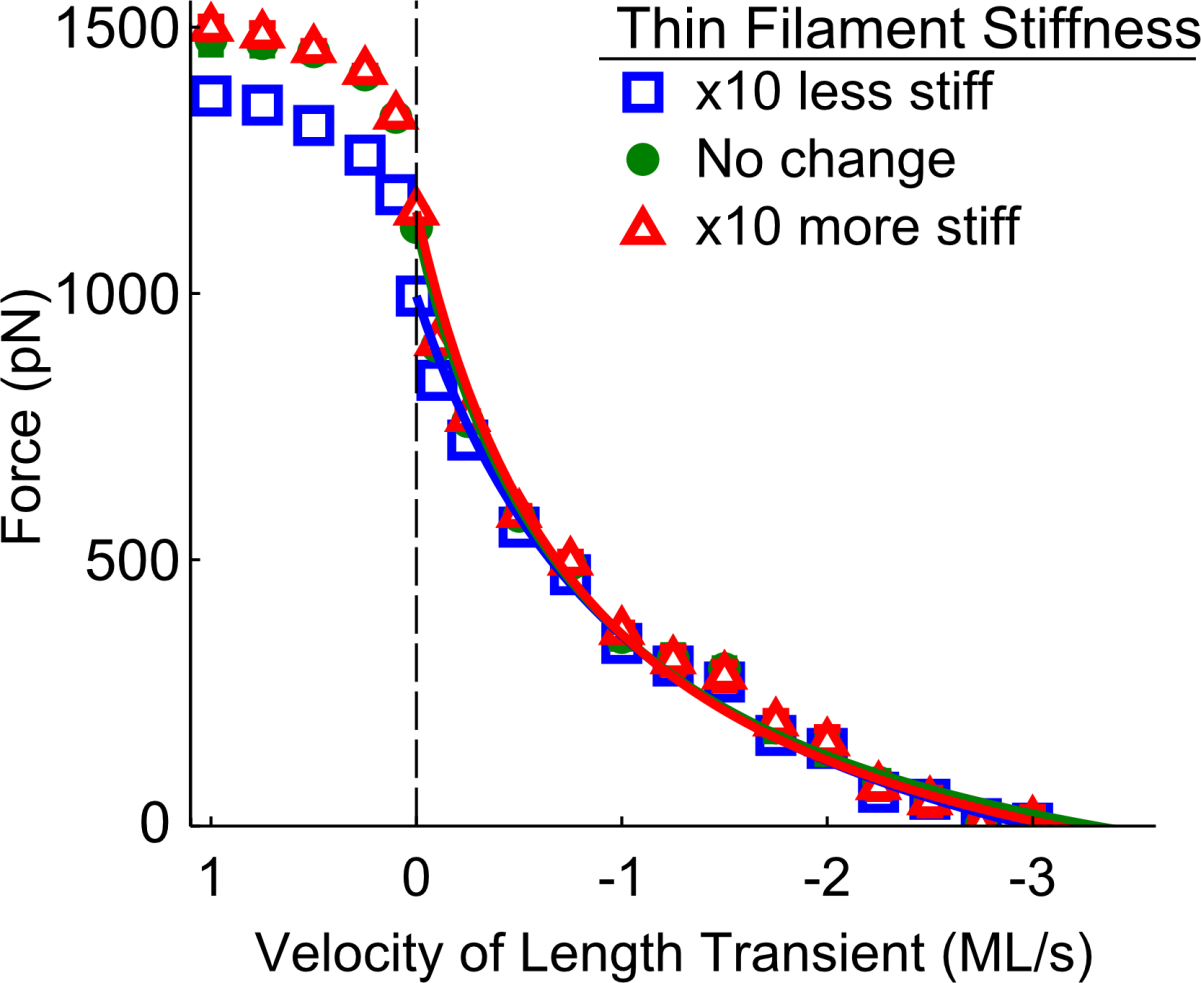
Velocity-dependent force measurements as a function of thin-filament stiffness. Decreasing thin-filament compliance by an order of magnitude resulted in lower tension during isometric contractions. However, increasing or decreasing the compliance of the thin-filament had minimal effects on the force-velocity relationship during sarcomere shortening.

In addition to XB stiffness, the stiffness of myosin and actin filaments was also adjusted to determine their potential contributions to the force-velocity relationships and power output. Increasing or decreasing myosin filament stiffness by an order of magnitude had an unremarkable effect on force production, cross-bridge binding, or ATP utilization (data not shown). Decreasing the stiffness of the actin filament by an order of magnitude resulted in decreased force production during isometric contractions, though these differences were negligible during sarcomere shortening (Fig 5). There was also little change in force production, cross-bridge binding, or ATP utilization when myosin and actin stiffness were altered simultaneously, except for the persistence of decreased force production during lengthening when the stiffness of both filaments was reduced by an order of magnitude (data not shown).

## Discussion

We performed this series of numerical experiments to investigate how force development, cross-bridge kinetics, and ATP utilization are influenced by sarcomere shortening and lengthening across a range of velocities. In addition, we examined how these parameters were affected by changes in cross-bridge and myofilament compliance. Our data show that the typical force-velocity relationship [2] involves robust velocity-dependent changes in cross-bridge number and ATPase activity, and that these changes are sensitive to cross-bridge compliance. These simulations reveal potential mechanical and biochemical optimizations that occur as sarcomeres generate power at different contraction velocities, and advance our understanding of the functional linkage between cross-bridge and whole tissue behavior.

According to the force-velocity relationship [2,3], the force generated by actively contracting muscle decreases as the shortening velocity increases until the maximal unloaded shortening velocity (*V*_*max*_) is reached. Our simulated force-velocity curves had profiles which were comparable to those obtained from empirical measurements, including a rapid rise in force during lengthening transients that plateaued at a value roughly 50% greater than isometric conditions (Fig 3A). The estimated *V*_*max*_ measured with default myofilament stiffness (3 pN/nm) was within the range of empirical values measured in mammalian skeletal muscle [38,39]. Increasing cross-bridge stiffness lowered maximal power output and shifted *V*_*opt*_ toward a slower velocity, and the opposite trend was observed when cross-bridge stiffness was decreased (Fig 4B). These data align with empirical data from in-vitro motility assays which show that increasing cross-bridge stiffness slows filament velocity [31], and suggest that modulation of cross-bridge stiffness may have a profound impact on the efficiency of power generation.

Force- and power-velocity relationships of muscle emerge primarily from cross-bridge properties within the sarcomere. Under unloaded conditions, the maximal shortening velocity is limited only by the cycling rate, particularly the ADP release rate [40]. Our data show that the ability of myosin to bind to actin is also directly affected by the contraction velocity. In agreement with estimates from fiber studies, cross-bridge binding decreased during high-velocity length transients [41], as binding may become unfavorable due to the speed at which actin-target sites pass unbound cross-bridges, and the increased strain on existing cross-bridges may forcibly detach them. However, we also found that changing sarcomere length at relatively slow velocities (both shortening and lengthening) augmented cross-bridge binding (Fig 3C). This increase in cross-bridge number likely follows from dynamic motion of the filaments, which increases the probability that the myosin head will interact with an available actin binding site [15,17,30]. The fraction of bound cross-bridges increased when cross-bridge stiffness was reduced (Fig 4C), suggesting that the increased flexibility of these bridges improves their ability to encounter and bind to an actin-target site, and that more flexible cross-bridges endure greater deformation before detaching. While more compliant cross-bridges produced less force under isometric conditions, their ability to remain bound at faster velocities allowed for relatively low forces to be maintained across a greater velocity range (Fig 4D). During shortening, the peak number of cross-bridges also occurred at a faster velocity, further evidence that the rightward shift in the power-velocity curve as stiffness decreases stems from increased cross-bridge binding (Fig 4C). Conversely, stiffer cross-bridges were more sensitive to changes in shortening velocity, which decreased force production over a smaller velocity range. Altogether, these data imply that cross-bridge stiffness plays a critical role in dictating the velocity range over which muscles produce power by modulating cross-bridge binding and force generation as shortening velocity changes.

Myosin regulatory light chain (RLC) phosphorylation status represents one of the principal modulators of actomyosin cross-bridge compliance in empirical studies [42], where RLC phosphorylation may increase the stiffness of the lever arm [43,44]. Fiber studies suggest that RLC phosphorylation increases the rate of transition from the weakly bound to strongly bound state, with little to no effect on the reverse-isomerization transition from strong to weak [45–47]. Motility assay data also suggests that RLC phosphorylation increases ATPase activity, with cross-bridge spending a larger fraction of the ATPase cycle bound to actin [31]. In combination, a faster transition from the weak to strongly bound state and higher duty ratio could increase the population of bound cross-bridges and augment force production as cross-bridge stiffness increases. These findings support our observation that increased cross-bridge stiffness increases the force per cross-bridge and decreases overall ATPase activity (Fig 4D and 4E), even though we did not see an increase in the fraction of bound cross-bridges as stiffness increased. This may be partially explained by a series of computational studies by Sheikh et al. (2012), showing that increased cross-bridge binding with light chain phosphorylation cannot be entirely attributed to stiffening of the lever arm. Instead, Sheikh et al. suggest that the negative charge from phosphorylation may repulse the thick filament and cause myosin heads to move away from the backbone, priming them for more probable binding to actin [43,44]. In our computational studies we only simulated the effect of cross-bridge stiffness without mimicking any changes in myosin head repulsion from the backbone, and cross-bridge binding increased with cross-bridge compliance. ATPase activity also increased with greater cross-bridge compliance, due to combined effects of more cross-bridge binding and faster cross-bridge cycling. Greater cross-bridge compliance may also permit more internal realignment of the neck-region of cross-bridge, where greater flexibility or extensibility could facilitate conformational transitions from one state to another. A potential consequence of this flexibility would be that more force goes into distortion of the neck and less force goes into tension propagation along the filaments, which leads to decreased force per cross-bridge and economy of contraction as cross-bridge compliance increases (Fig 4D and 4F).

According to recent empirical studies, the myosin ATPase rate may be reduced when the lever arm is strained [48,49], and stiffening of the lever arm may increase the strain sensitivity. As the duty cycle increases, cross-bridges spend a greater portion of their cycle bound to myosin in a force-generating state, which could limit the sliding velocity. While decreased sliding velocity is observed with light chain phosphorylation in in-vitro motility assays, the decreased velocity was still faster than was predicated for the given change in duty cycle, possibly due to an increase in the unitary step size [31]. We calculated force during a fixed velocity, and found that force continued to be maintained at a faster shortening velocity when cross-bridge stiffness was decreased, but with less force per cross-bridge (Fig 4D). Though we observed a decrease in overall sarcomere ATPase activity when cross-bridge compliance was reduced (Fig 4E), this was primarily due to a decrease in the total fraction of bound cross-bridges.

We were surprised to see that the force-velocity relationship was relatively insensitive to myofilament compliance. Increasing or decreasing thick-filament compliance had little or no observable effect on the force- or power-velocity relationships (data not shown), and while some variance was observed with altered thin-filament compliance, these changes disappeared during shortening transients (Fig 5). In addition, the effects of myofilament compliance on force development, cross-bridge kinetics, and ATP utilization during shortening and lengthening transients were unremarkable. Numerous computational simulations of ‘isometric’ muscle contraction suggest that myofilament compliance significantly influences force development, cooperativity of the force-pCa relationship, and the rate of force development [15–18,20,30]. In combination with these prior studies, our new simulations suggest that the role of myofilament compliance appears to be more influential during isometric contractions by allowing for small movement within the lattice, but that this movement is overshadowed by the large changes imposed during sarcomere shortening. This indicates that cross-bridge stiffness may play an important role in modulating dynamic contraction (i.e. the force- and power-velocity relationships), while myofilament stiffness plays a more prominent role during isometric contraction.

Both the amount of force produced and the energy consumed during normal physiological muscle movement fluctuates with muscle length [2,50]. This can be partially explained by changes in filament overlap within the sarcomeres, as a greater number of bound cross-bridges can produce more force and consume more energy. However, the picture of cross-bridge kinetics and their contribution to contractile efficiency of the sarcomere is complicated during dynamic motion, as the force generation, cycling rate, and binding-site orientation of individual cross-bridges is in flux. Our observations illustrate the complex relationship between the velocity of length transients and cross-bridge properties. In particular, our predictions show that the increased energy consumption that is measured as functional muscle shortens is likely due not only to faster cross-bridge cycling, but also to a velocity-dependent change in the fraction of bound myosin heads.

## Conclusions

The efficiency of muscle force production decreases as shortening velocity increases, an observation first described by Wallace Fenn in 1923 using frog sartorius muscle. However, the molecular mechanisms that contribute to this velocity-dependent change in contractile efficiency are difficult to measure empirically. We used spatially-explicit, multi-filament models of muscle contraction within a half-sarcomere to investigate potential mechanisms that coordinate myosin force production and energy utilization during lengthening and shortening. We found that the fraction of bound cross-bridges increased during slow-velocity concentric and eccentric contractions, but that ATP utilization increased during shortening transients due to faster cross-bridge cycling. The fraction of cross-bridges bound and ATPase activity also both increased in models with reduced cross-bridge stiffness while the force per cross-bridge decreased, resulting in reduced economy of force production within the sarcomere. These findings improve our understanding of how contractile efficiency and power output are modulated throughout dynamic contractions to facilitate muscle movement and locomotion.

## Methods

Building upon a spatially-explicit computational model of the half-sarcomere [20], we simulated Ca^2+^-activated muscle contraction during lengthening, shortening, and isometric conditions. Prior model development and validation primarily focused on matching isometric tension values, Ca^2+^-sensitivity of contraction, and the associated rates of tension development and ATPase from rabbit fast-twitch muscle [51–53].

### Myofilament geometry

In brief, these models comprise 4 thick- and 8 thin-filaments represented as a network of linear elastic springs. Each thick-filament is composed of 61 spring elements linked together in series at ‘nodes’. At each node three myosin head-pairs extend radially from each thick-filament node with 120° between each myosin. From one node to the next the trio of heads rotates 40° to reflect the three-start helical pitch of myosin crowns in vertebrate striated muscle [54–56]. Each thin-filament is composed of 91 spring elements linked together in series, and represents the helical pitch of vertebrate thin-filaments (rotating 180° every 37.7 nm, [57]). Thin-filament regulatory unit activation span was set at 50 nm for all simulations, representing activation of ~9 actin monomers along one actin helix upon Ca^2+^-activation of troponin and subsequent tropomyosin movement to make thin-filament sites available for myosin cross-bridge binding [20]. Thick-filaments were ~860 nm long (= 60 nodes x 14.3 nm) and thin-filaments were ~1110 nm (= 90 nodes x 12.3 nm), with the resting half-sarcomere length set to 1200 nm during simulations of isometric contraction prior to any lengthening or shortening transients (representing a sarcomere length of 2.4 μm and almost complete thick-to-thin filament overlap as would be expected at or near the plateau of the length-tension relationship). Potential actin-myosin cross-bridge interaction between two adjacent filaments can occur if they are properly aligned longitudinally (depending upon distortion-dependent cross-bridge kinetics, [20]) and rotationally (±20°, [58]). Periodic boundary conditions along the length of the half-sarcomere remove any edge effects along the longitudinal boundary of the simulation and enables predictions of muscle contraction from a finite number of filaments to represent a subsection myofilament lattice space.

### Kinetics

Thin-filament activation represents three states (Fig 1): TF1) Ca^2+^ is not bound to troponin and the actin-target sites along the thin-filament are unavailable for myosin binding, TF2) Ca^2+^ is bound to troponin, but the there is no movement of tropomyosin so the binding sites remain unavailable, and TF3) Ca^2+^ is bound to troponin and the movement of tropomyosin reveals an available myosin biding site. Cross-bridge cycling is also represented as a three-state model: XB1) Myosin is unbound from actin, XB2) myosin is bound to actin as a low-force bearing, pre-power stroke cross-bridge, and 3) myosin is bound to actin as a high-force bearing, post-power stroke cross-bridge. This kinetic scheme includes cooperative activation of thin-filaments from neighboring thin-filament regulatory units and bound cross-bridges and distortion-dependent cross-bridge transition rates identical to our previous models [20,58]. Monte Carlo algorithms drive kinetic state transitions for both thin-filament activation and cross-bridge binding, using a 1 ms time-step for all simulations.

### Myofilament and cross-bridge compliance

Unless otherwise noted, spring elements for the thick- and thin-filaments were 6060 and 5230 pN nm^-1^ for resting (unstrained) elements of length 14.3 and 12.3 nm, respectively. Default cross-bridge stiffness was set at 3 pN nm^-1^, consistent with the parameter value used in our previous studies and estimates from cellular experiments [59–61]. We also included a non-linear spring element to represent titin and simulate sarcomere length-dependent changes in passive tension during shortening and lengthening transients. The non-linear relationship between passive tension and half-sarcomere length was solved using the estimated length change imposed and a titin stiffness coefficient as described by Campbell 2009 [30,62]:

$$F_{pas}=\sigma *exp(\frac{x_{hs}-x_{offset}}{L})$$ (1)

where F_pas_ is the passive force produced by a length change (x_offset_) of a half-sarcomere with a given length (x_hs_). Values for σ (0.4037 N m^-2^) and L (200.2 nm) were found by curve fitting. At each time-step, titin stiffness was updated to provide the appropriate amount of passive force for a given change in sarcomere length. Potential interactions between titin and the myofilaments were not modeled.

### Shortening and lengthening protocol

Lengthening and shortening transients were achieved by changing the M-line position with respect to the Z-line (effectively altering half-sarcomere length without changing any myofilament geometry parameters). Concentric and eccentric contractions were simulated by shortening 100 nm, then held for 250 ms to achieve a stable isometric contraction, followed by re-lengthening to the original half-sarcomere length (Fig 2). This protocol ensured similar changes in thick-to-thin filament overlap and relative effects on the potential number of actin-myosin cross-bridge interactions for all simulations. Each pair of shortening and lengthening transients was performed as an individual simulation run at a particular velocity with a minimum of 25 run iterations. The complete range of velocity data were combined to create the force-velocity relationship. Force-velocity curves were fit as described by Hill (1938):

$$\left(T+a\right)\left(V+b\right)=\left(T_{0}+a\right)b$$ (2)

where T is tension, V is velocity, and T_0_ is isometric tension.
